# Commentary: Preventive Treatments for Psychosis: Umbrella Review (Just the Evidence)

**DOI:** 10.3389/fpsyt.2020.00488

**Published:** 2020-05-27

**Authors:** Barnaby Nelson, G. Paul Amminger, Andrew Thompson, Stephen J. Wood, Alison R. Yung, Patrick D. McGorry

**Affiliations:** ^1^Orygen, Parkville, VIC, Australia; ^2^Centre for Youth Mental Health, The University of Melbourne, Parkville, VIC, Australia; ^3^School of Psychology, University of Birmingham, Birmingham, United Kingdom

**Keywords:** psychosis, preventive treatment, high risk, schizophrenia, psychological therapy

It is striking that the field of intervention for young people at high risk of psychotic disorders has progressed to the point that an umbrella review of evidence (a review of meta-analyses) is even feasible. This is testament to the dedication of a number of research groups internationally and the spread of early intervention services. Fusar-Poli and colleagues (1) conclude, based on their review of 7 meta analyses made up of 20 intervention trials, that there is no evidence to favor any preventive intervention over any other (or control condition) for improving clinical outcomes in the high-risk clinical population and that caution is required when making clinical recommendations for this group. While much in the review is balanced commentary, we suggest that this “take home” message is a partisan *interpretation* of the current evidence base, in contrast to what is implied by the review’s title (“just the evidence”).

We base this argument on two key points:

1. The fact that no single treatment has been identified as being superior to others in improving clinical outcomes should not be seen as a disappointment or failure of the field. The “dodo bird verdict” (so-named after the dodo bird in *Alice in Wonderland* (1865), who declares after a running contest that “everyone has won and all must have prizes!”), i.e., a number of interventions have the same effect, is not uncommon in many mental and physical disorders. Indeed, this has been a highly active area of discussion in psychotherapy research over the last several decades (2). As discussed in that literature, the “dodo bird verdict” may partly be a product of group-level statistics (2). That is, individual differences in response to treatments (e.g., person X responding to CBT, person Y responding to omega-3 fatty acids) are cancelled out by aggregating across individuals in group-level analysis, with sample sizes not currently sufficient to confidently identify sub-group therapeutic response. We agree with the review’s authors that the field now needs to parse heterogeneity and identify sub-groups who are more likely to respond to particular therapies compared to others. In fact, this is what we have recently done in a secondary analysis of our omega-3 intervention trial in this clinical group, identifying that omega-3 supplementation was particularly effective for patients with low omega-3 fatty acid levels at study entry and that clinical improvement was associated with omega-3 fatty acid increase during the trial. The NEURAPRO biomarker analysis also showed that higher omega-3 baseline levels predicted better clinical outcomes independent of further supplementation, highlighting the importance of biomarker guided targeted intervention (3). Pursuing this “precision medicine” avenue in future trials requires large samples, international consortia-based groups, and replication trials. Another (not mutually exclusive) approach, which might be more feasible given the current state of knowledge, is staged treatment designs, which adjust treatment based on treatment response/clinical progress and dynamic prediction of likely outcomes. This is the approach we have adopted in our current sequential intervention trial (SMART) in this clinical population (4).
  The apparent equivalence of various therapies should not be seen as clinically meaningless, as suggested in the review. Being able to offer a range of treatments, and not yet being able to fine tune/personalize this treatment given the current state of knowledge, puts a strong emphasis on the role of patient preference and shared decision making. This approach to treatment, with patient-directed care and a strong collaborative approach, has been found to increase compliance and possibly enhance the effectiveness of treatment (5–7).
  Another way of viewing the “dodo bird verdict” is that it points toward possible potent *shared factors* in treatment, which has not been adequately recognised in the field to date. Obvious candidates are the role of hope for clinical improvement and risk reduction (8), therapeutic alliance, practical case management, and early intervention service milieu (9, 10). Additionally, the possible therapeutic impact of the alliance formed between a young person and research interviewer (via regularity of contact, enquiring as to current mental state, contextual factors, etc.) and the positive impact of self-monitoring that research participation entails (*via* research interviews, ecological momentary assessment, etc.) should not be underestimated (11).
2. This leads us to the possible contribution of improved standard treatment in clinical high risk services. Imagine if a commentator had remarked in the mid 1990s, when the clinical high risk field was starting out, that a mark of success for the field would be seeing transition to psychosis rates in this clinical population drop to about 10% (from an initial 30%–40%) by 2020, including in those who receive standard clinical care. Some may have seriously doubted the possibility of ever achieving this, but few would have disagreed that this would represent a major health success. While we agree with the review’s authors about the importance of clarifying next steps for the field, we should recognise that substantial progress has been made in “standard treatment” in early intervention services for this clinical population and therefore effective interventions are perhaps “hiding in plain sight”.
  Standard treatment for this clinical population is an active psychosocial intervention and has been refined over the years (i.e., it is not a fixed entity), particularly in specialised clinical research services where most of these intervention trials have been conducted. Standard treatment as a comparison condition might have become more effective in recent trials, coupled with the observed rise in placebo response (12), which may effectively have introduced a ceiling effect for finding additional benefit of specific trial interventions. While it is challenging to distil the effective ingredients of standard treatment (some possibilities are listed above), we recently conducted a file audit of patients who received standard treatment in our high risk clinic at Orygen to assess this issue (13). Findings indicated that increases in the provision of CBT, problem solving therapy, and duration of treatment modulated the relationship between year of entry and transition risk. In other words, increases in these treatment components may have contributed to the reduction in psychosis risk in this clinical population over time. Also of note is that standard treatment generally includes the treatment of comorbidities (e.g., through the use of evidence-based psychotherapy and antidepressant medication) (4, 14) which may reduce psychosis risk, as well as psychoeducation and enhancing coping skills, which may in themselves be effective in dealing with stress as a trigger of psychotic symptoms (15), consistent with the stress-vulnerability of psychosis (16).
  Another example of how ‘standard’ treatment may have shifted over time is with regard to duration of transdiagnostic symptoms prior to receiving treatment. This variable has been identified as one of the most potent predictors of transition risk, regardless of whether the patient receives standard care or controlled trial treatment (**Figure 1**). Patients with a short duration of symptoms are more highly represented in recent cohorts (17, 18). **Figure 1** shows that the rate of psychosis onset in patients with a shorter duration of symptomatology prior to treatment (<1 year) is significantly lower than those with a longer duration of symptoms prior to treatment, even when followed over a 10-year period [with the length of this follow up period showing that this is not a lead time bias effect (17)]. This further illustrates how “standard” treatment may have shifted over time (toward *earlier* intervention), resulting in improved clinical outcomes. While sampling issues and recruitment strategies may also have contributed to the reduced transition risk in this clinical population (17, 19–24), the evidence for this is not strong, with baseline data in fact indicating that clinical severity has not altered substantially in clinical high risk patients over the decades (19). In contrast, the observations and data flagged above indicate that structural and content changes to standard treatment may be strong contributors to the reduced psychosis transition rates. In our view, the fact that this has not been sufficiently recognised to date may be partly due to the difficulty of systematically capturing standard treatment changes, but also partly due to the fixation in the psychosis research field on biomarkers and biotherapies, as though these represented the only real signs of progress (25). This approach disregards the potency of comprehensive psychosocial care, possibly particularly in young people in the early stages of disorder.
  Finally, a point of clarification. The review misinterprets a previous article of ours published in *Frontiers in Psychiatry* (26), specifically our use of the phrase “second order issue”. Rather than referring to transition to full-threshold psychosis as an outcome in clinical high risk intervention studies as a “second order issue”, as the review claims, we were referring to the question of *which specific trial treatment* is the most effective as being a second order issue. We are certainly not advocating that transition to psychosis should be marginalised as an outcome of interest, as the review suggests that we were.

**Figure 1:**
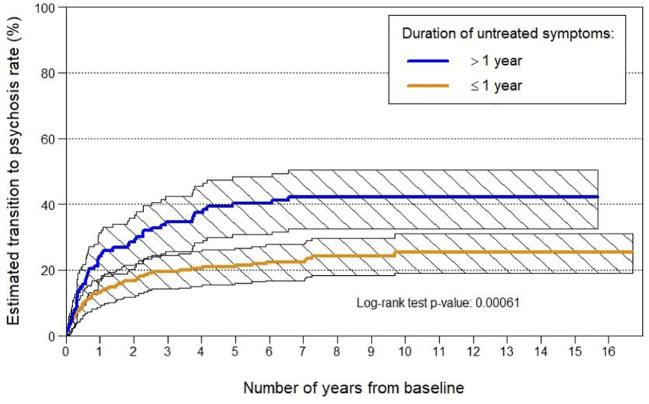
Transition to psychosis rates by duration of symptoms prior to entry to Orygen’s ‘ultra high risk’ for psychosis clinic.

## Author Contributions

All authors contributed to the content of this paper.

## Conflict of Interest

The authors declare that the research was conducted in the absence of any commercial or financial relationships that could be construed as a potential conflict of interest.

## References

[1] Fusar-Poli P Davies C Solmi M Brondino N De Micheli A Kotlicka-Antczak M Preventive Treatments for Psychosis: Umbrella Review (Just the Evidence). Front Psychiatry (2019) 10:764. 10.3389/fpsyt.2019.00764 31920732 PMC6917652

[2] Wampold BE Imel ZE The great psychotherapy debate: The evidence for what makes psychotherapy work. 2nd edition London: Routledge (2015).

[3] Amminger GP Nelson B Markulev C Yuen HP Schäfer MR Berger M The NEURAPRO Biomarker Analysis: Long-Chain Omega-3 Fatty Acids Improve 6-Month and 12-Month Outcomes in Youths at Ultra-High Risk for Psychosis. Biol Psychiatry (2020) 87(3):243–52. 10.1016/j.biopsych.2019.08.030 31690495

[4] Nelson B Amminger GP Yuen HP Staged Treatment in Early Psychosis: A sequential multiple assignment randomised trial of interventions for ultra high risk of psychosis patients. Early Interv Psychiatry (2018) 12(3):292–306. 10.1111/eip.12459 28719151 PMC6054879

[5] Shay LA Lafata JE Where is the evidence? A systematic review of shared decision making and patient outcomes. Med Decis Making (2015) 35(1):114–31. 10.1177/0272989X14551638 PMC4270851 25351843

[6] Nott J McIntosh A Taube C Taylor M Shared decision-making in psychiatry: a study of patient attitudes. Australas Psychiatry (2018) 26(5):478–81. 10.1177/1039856218758562 29457470

[7] Slade M Implementing shared decision making in routine mental health care. World Psychiatry (2017) 16(2):146–53. 10.1002/wps.20412 PMC5428178 28498575

[8] Bressan RA Iacoponi E Candido de Assis J Shergill SS Hope is a therapeutic tool. BMJ (2017) 359:j5469. 10.1136/bmj.j5469 29237595

[9] McGorry PD Killackey E Yung A Early intervention in psychosis: concepts, evidence and future directions. World Psychiatry (2008) 7(3):148–56. 10.1002/j.2051-5545.2008.tb00182.x PMC2559918 18836582

[10] Correll CU Galling B Pawar A Krivko A Bonetto C Ruggeri M Comparison of Early Intervention Services vs Treatment as Usual for Early-Phase Psychosis: A Systematic Review, Meta-analysis, and Meta-regression. JAMA Psychiatry (2018) 75(6):555–65. 10.1001/jamapsychiatry.2018.0623 PMC6137532 29800949

[11] Korotitsch WJ Nelson-Gray RO An overview of self-monitoring research in assessment and treatment. psychol Assess (1999) 11(4):415–25. 10.1037/1040-3590.11.4.415

[12] van Os J Guloksuz S Vijn TW Hafkenscheid A Delespaul P The evidence-based group-level symptom-reduction model as the organizing principle for mental health care: time for change? World Psychiatry (2019) 18(1):88–96. 10.1002/wps.20609 30600612 PMC6313681

[13] Formica M Phillips LJ Hartmann JA Has improved treatment contributed to the declining rate of transition to psychosis in ultra-high-risk cohorts? Schizophr Res (2020). Under review. 10.1016/j.schres.2020.04.028 32402606

[14] Schmidt SJ Schultze-Lutter F Schimmelmann BG Maric NP Salokangas RK Riecher-Rossler A EPA guidance on the early intervention in clinical high risk states of psychoses. Eur Psychiatry (2015) 30(3):388–404. 25749390 10.1016/j.eurpsy.2015.01.013

[15] Schmidt SJ Schultze-Lutter F Schimmelmann BG EPA guidance on the early intervention in clinical high risk states of psychoses. Eur Psychiatry (2015) 30(3):388–404. 10.1016/j.eurpsy.2015.01.013 25749390

[16] Pruessner M Cullen AE Aas M Walker EF The neural diathesis-stress model of schizophrenia revisited: An update on recent findings considering illness stage and neurobiological and methodological complexities. Neurosci Biobehav Rev (2017) 73:191–218. 10.1016/j.neubiorev.2016.12.013 27993603

[17] Nelson B Yuen HP Lin A Wood SJ McGorry PD Hartmann JA Further examination of the reducing transition rate in ultra high risk for psychosis samples: The possible role of earlier intervention. Schizophr Res (2016) 174(1-3):43–9. 10.1016/j.schres.2016.04.040 27173977

[18] Nelson B Yuen HP Wood SJ Lin A Spiliotacopoulos D Bruxner A Long-term follow-up of a group at ultra high risk (“prodromal”) for psychosis: the PACE 400 study. JAMA Psychiatry (2013) 70(8):793–802. 10.1001/jamapsychiatry.2013.1270 23739772

[19] Hartmann JA Yuen HP McGorry PD Yung AR Lin A Wood SJ Declining transition rates to psychotic disorder in “ultra-high risk” clients: Investigation of a dilution effect. Schizophr Res (2016) 170(1):130–6. 10.1016/j.schres.2015.11.026 26673973

[20] Wiltink S Velthorst E Nelson B McGorry PM Yung AR Declining transition rates to psychosis: the contribution of potential changes in referral pathways to an ultra-high-risk service. Early Interv Psychiatry (2015) 9(3):200–6. 10.1111/eip.12105 24224963

[21] Fusar-Poli P Schultze-Lutter F Cappucciati M The Dark Side of the Moon: Meta-analytical Impact of Recruitment Strategies on Risk Enrichment in the Clinical High Risk State for Psychosis. Schizophr Bull (2016) 42(3):732–43. 10.1093/schbul/sbv162 PMC4838090 26591006

[22] Yung AR Yuen HP Berger G Francey S Hung TC Nelson B Declining transition rate in ultra high risk (prodromal) services: dilution or reduction of risk? Schizophr Bull (2007) 33(3):673–81. 10.1093/schbul/sbm015 PMC2526154 17404389

[23] Fusar-Poli P Why ultra high risk criteria for psychosis prediction do not work well outside clinical samples and what to do about it. World Psychiatry (2017) 16(2):212–3. 10.1002/wps.20405 PMC5428173 28498578

[24] Fusar-Poli P Palombini E Davies C Oliver D Bonoldi I Ramella-Cravaro V Why transition risk to psychosis is not declining at the OASIS ultra high risk service: The hidden role of stable pretest risk enrichment. Schizophr Res (2018) 192:385–90. 10.1016/j.schres.2017.06.015 28734908

[25] Kingdon D Why hasn’t neuroscience delivered for psychiatry? BJPsych Bull, (2020). 1–3. 10.1192/bjb.2019.87

[26] Nelson B Amminger GP McGorry PD Recent Meta-Analyses in the Clinical High Risk for Psychosis Population: Clinical Interpretation of Findings and Suggestions for Future Research. Front Psychiatry (2018) 9:502. 10.3389/fpsyt.2018.00502 30369889 PMC6194228

